# “Secreted in Xylem” Genes (SIX Genes): Relationship to the Aggressiveness of *Fusarium oxysporum* f. sp. *albedinis*

**DOI:** 10.3390/plants14111721

**Published:** 2025-06-05

**Authors:** Abdelhi Dihazi, Youness Jouihri, Ahmed Tadlaoui-Ouafi, Mohamed Najib Alfeddy, Cherkaoui El Modafar, Hassan Dihazi, Abdellatif El Meziane, Mohammad Sayari, Fouad Daayf

**Affiliations:** 1 Centre d’Agrobiotechnologie et Bioingénierie, Unité de Recherche Labellisée CNRST (URL-CNRST 05), Universite Cadi Ayyad, Marrakech 40000, Morocco; younessjouihri027@gmail.com (Y.J.); a.tadlaouiouafi@uca.ac.ma (A.T.-O.); elmodafar@uca.ac.ma (C.E.M.); 2 Laboratory of Phyto-Bacteriology, Research Unit of Plant Protection, National Institute of Agricultural Research (INRA), Marrakech 40000, Morocco; mohamednajib.alfeddy@inra.ma; 3 Clinic for Nephrology and Rheumatology, University Medical Center Göttingen 1, 37075 Göttingen, Germany; dihazi@med.uni-goettingen.de; 4 Department of Plant Science, University of Manitoba, Winnipeg, MB R3T 2N2, Canada; mohammad.sayari@umanitoba.ca

**Keywords:** *Fusarium oxysporum albedinis*, Bayoud disease, aggressiveness, secretion, xylem

## Abstract

*Fusarium oxysporum* f. sp. *Albedinis* (*Foa*) is the causal agent of Bayoud disease, responsible for the loss of 75% of date palm trees in Morocco and posing a threat to its cultivation across North Africa. This study examined ten *Foa* isolated from various Moroccan locations for the presence of the transposable element *Fot1* and the distribution of “Secreted in Xylem” (SIX) genes. Pathogenicity assays on date palm seedlings revealed varying levels of aggressiveness among isolates, with a positive correlation between aggressiveness and SIX gene count. Highly aggressive isolates harbored 9–12 SIX genes, while hypo-aggressive and moderately aggressive isolates carried 0–6. SIX2, SIX6, SIX7, SIX11, SIX12, and SIX13 were differently dispersed among aggressive isolates, whereas SIX12 and SIX13 were present in all aggressive isolates, suggesting their potential role in virulence. This study is the first to highlight a correlation between *Foa* aggressiveness and SIX gene distribution, providing a foundation for future functional analyses to elucidate their role in pathogenicity.

## 1. Introduction

Date production is the primary economic activity for the population living in Moroccan oases, accounting for 60% of farmers’ income in those regions [1]. However, Bayoud disease, caused by *Fusarium oxysporum* f. sp. *albedinis* (*Foa*), led to the loss of over 12 million (>75%) date palm trees [2], resulting in significant economic, ecological, and social impacts [3]. In neighboring Algeria, the disease has caused the loss of over three million date palm trees [4] and remains the heaviest threat to date palm cultivation in the region.

This disease’s symptoms appear first on the leaves, which begin to fade and take on a particular appearance resembling a wet feather, before completely drying out and wilting. The pathogen infects the roots first and strongly colonizes them, then progresses upwards into the vascular stele to reach the upper leaves. It induces severe host cell alterations, including cell wall degradation and vesicle formation, leading to date palm death [5]. Once date palm trees are infected by *Foa*, no effective treatment can be prescribed, especially when the pathogen is highly aggressive [6]. Some farmers reported date palm trees that could withstand Bayoud disease after symptoms had appeared, eventually returning to a healthier state. The mechanisms behind these phenotypes and changes in disease expression are not understood yet. Few studies investigated the difference between aggressive and hypo-aggressive isolates in *Foa* [6,7]. We previously reported many differences in protein expression between two isolates that differ in aggressiveness [6], but no work has addressed the question at the gene level. Moreover, the virulence in *F. oxysporum* species was reportedly associated with accessory chromosomes [8], which are also involved in host specificity [9]. These can transfer horizontally between strains during their vegetative growth [8,10]. In *F. oxysporum* f. sp. *lycopersici*, Ma et al. (2010) reported eleven core chromosomes responsible for the saprophytic growth in addition to four accessory chromosomes [8]. The transfer of accessory chromosome 14 into *F. oxysporum* f. sp. *lycopersici* converted a non-pathogenic strain into a pathogenic one in tomato plants. Furthermore, accessory chromosomes were reported to be rich in transposable elements and to carry genes responsible for pathogenicity [8,10,11,12]. Fourteen genes named *SIX* (secreted in xylem genes) and designated from *SIX1* to *SIX14* have been identified in *F. oxysporum* f. sp.*lycopersici* [11,13,14]. They encode for small proteins rich in cysteine [11] and are expressed in the xylem during plant colonization. These proteins, labeled as effectors, could distinguish between pathogenic and non-pathogenic forms of the pathogen [15].

The deletion of some *SIX* genes in *F. oxysporum* f. sp. *lycopersici* significantly reduced the pathogenicity against tomato plants [14,16,17,18]. Homologs of *SIX* genes were largely reported in other *F. oxysporum* strains [16,19,20,21,22,23,24,25,26]. The difference in their distribution in *F. oxysporum* was linked to pathogenicity [19,21], i.e., the profile of *SIX* genes distinguished between highly and weakly virulent isolates of *F. oxysporum* f. sp. *cepae* [21]. Similar results were reported for *F. oxysporum* f. sp. *cubense* infecting banana [27] and *F. oxysporum* f. sp. c*onglutinans* infecting Brassicas [28].

In *F. oxysporum* f. sp. *albedinis*, variability in aggressiveness has been described [6,7], but little is known about the genetic basis of such variability. Based on an in silico sequence analysis of *Foa*, Rafiqi et al. (2022) reported four *SIX* effectors: two similar to *SIX1*, one similar to *SIX9*, and one similar to *SIX11* [29]. Based on previous work reported in other *Fusarium* species [22], they associated *SIX9* and *SIX11* with pathogenicity. However, this has not been proven because the *SIX* genes responsible for virulence vary from one format to another.

The objectives of this study were (i) to compare the aggressiveness of 10 *Foa* isolates from three major date-palm-producing regions in Morocco (Zagora, Errachidia, and the Taznakht–Tata axis); (ii) to screen for and compare the presence of *SIX* gene homologs in these isolates; and (iii) to investigate whether such *SIX* genes are associated with the aggressiveness of the isolates carrying them. This study is meant to set the basis for future functional analyses of selected *SIX* genes that show a strong association with pathogenicity. Understanding these relationships is crucial for developing effective disease management strategies and improving the resistance of date palm cultivars to Bayoud disease.

## 2. Material and Methods

### 2.1. Foa Isolation

The *Foa* isolates were collected from different regions of Morocco, including Zagora, Errachidia, and the Taznakht–Tata axis (Supplementary Figure S1). Each isolate was labeled based on the name of the locality from which it was obtained.

The rachis from infected date palm leaves were cut into small pieces, surface-sterilized with 40% sodium hypochlorite for 5 min and 70% ethanol for 5 min and then rinsed and incubated at 25 °C on potato dextrose agar (PDA) medium supplemented with chloramphenicol (50 mg/mL).

Once the *Foa* mycelium grew, it was transferred to fresh PDA plates multiple times until a pure isolate was obtained. Subsequently, a *Foa* suspension was prepared using sterilized water and incubated on a new PDA medium to obtain a single-spore culture.

### 2.2. DNA Extraction from Foa

DNA was extracted from 7-day-old single-spore cultures grown on PDA, following the method of Gontia-Mishra et al. (2014) [30], with slight modifications. Briefly, mycelia (200 mg) were harvested using a sterilized scalpel by scraping the surface of the plates, then mixed with fine sand (100 mg) and ground in 1 mL of pre-warmed (65 °C) CTAB buffer. The extracts were incubated at 65 °C for 1 h after adding proteinase K (50 μg/mL), followed by the addition of β-mercaptoethanol (1%). After incubation, the extracts were centrifuged at 10,000 rpm for 10 min. The supernatant was mixed with an equal volume of chloroform–isoamyl alcohol (24:1) and centrifuged at 10,000 rpm for 10 min. The upper aqueous phase (500 µL) was collected, treated with 2.5 µL RNase (10 mg/mL), and incubated at 37 °C for one hour. Next, 50 µL of 3 M sodium acetate was added, followed by gentle mixing with an equal volume of chilled isopropanol. The samples were then incubated at −20 °C for one hour. After centrifugation at 13,000 rpm for 20 min, the supernatant was removed, and the DNA pellet was washed with 1 mL of 70% ethanol. Final centrifugation at 13,000 rpm for 15 min was performed, the ethanol was discarded, and the DNA was resuspended in 50 µL TE (Tris-HCl, EDTA, Invitrogen Thermo Scientific Chemicals, Mount Prospec, IL, USA) buffer.

### 2.3. Molecular Detection of Foa

To confirm that the 10 isolates were *Foa*, PCR was performed targeting the transposable element *Fot1* using specific primer pair TL3-FOA28 (FOA28: ATCCCCGTAAAGCCCTGAAGC and TL3: GGTCGTCCGCAGAGTATACCGGC). These primers generate a 400 bp amplicon exclusively with *F. oxysporum* f. sp. *albedinis* [31,32]. This assay was deemed sufficient to distinguish *Foa* from other saprophytic and pathogenic isolates of *Fusarium* isolates [31]. PCR reactions were carried out in a 25 µL reaction volume [31]. The reaction mixture contained 1 µL of genomic DNA, MgCl_2_ (1.5 mM), dNTP (0.4 mM), 1 unit of *Taq* DNA polymerase (Invitrogen, Thermo Fisher Scientific, Van Allen Way, Carlsbad CA, USA), 2.5 µL of 10X reaction buffer, and 0.5 µM of each primer. PCR was performed in a thermocycler (MJ Research, Inc., Waltham, MA, USA) under the following conditions: initial denaturation at 94 °C for 3 min, followed by 35 cycles of 30 s at 94 °C (denaturation), 30 s at 62 °C (annealing), and 1 min at 72 °C (extension), with a final extension step at 72 °C for 10 min. A negative control (no DNA template) was included in all PCR reactions. PCR products (10 µL) were separated by electrophoresis on a 2% agarose gel in TBE (Tris-borate EDTA) buffer, and DNA was visualized using ethidium bromide staining.

### 2.4. Aggressiveness Tests

All ten isolates produced a 400 bp amplified product using specific primers and were tested for aggressiveness. They were incubated on PDA for one week, after which conidia were collected in sterilized water and adjusted to 10^6^ conidia/mL, to be used as inoculum. Date palm seedlings were grown from seeds of cultivar Boufeggous (BFG), which is highly susceptible to Bayoud disease. The seeds were sterilized with sodium hypochlorite (20%) for 20 min, germinated on humidified peat at 28 °C, and transferred to a greenhouse under a 16 h light photoperiod. The seedlings were regularly irrigated. Twelve-month-old seedlings were removed from the soil and washed with tap water. Each seedling was then inoculated with 10 μL of the conidial suspension (10^6^ conidia/mL) using a sterile syringe. Ten seedlings per isolate were used, whereas the control group of 10 seedlings was injected with sterilized water. The plants were placed in 500 mL flasks containing Hoagland solution (Supplementary Figure S2) and maintained in a greenhouse at 30 °C. The experiment was carried out in two independent trials. Disease severity was assessed one, two, and three months post-inoculation by recording the number of wilted leaves (disease incidence) and the number of completely collapsed plants per isolate (disease severity). Each isolate was classified as hypoaggressive, moderately aggressive, or aggressive if the mortality rate at three months post-inoculation was 0%, between 0% and 50%, or more than 50%, respectively.

To confirm that the symptoms observed in date palm seedlings were caused by *Fusarium oxysporum* f. sp. *albedinis (Foa*), the pathogen was re-isolated from symptomatic plants three months post-inoculation. Five small leaf fragments from each plant were surface-sterilized in 20% sodium hypochlorite for 2 min, rinsed in sterile distilled water, and placed on PDA plates incubated at 28 °C. Emerging fungal colonies were sub-cultured, and isolates were identified based on morphological characteristics (colony color, growth rate, and conidial shape) consistent with the original *Foa* isolates. Identity was further confirmed by PCR using transposable element *Fot1*. To fulfill Koch’s postulates, re-isolated strains were used to inoculate healthy date palm seedlings, which subsequently developed similar wilt symptoms, confirming the pathogenicity of the isolates.

Multiple correspondence analysis (MCA) was performed to investigate associations between *Fusarium oxysporum* f. sp. *albedinis* isolate aggressiveness and the presence of various *SIX* genes. This analysis was conducted using IBM SPSS Statistics software Version 21.

Furthermore, hierarchical clustering was carried out using the average linkage method, which computes the average distance between all pairs of objects from different clusters. The analysis was conducted with IBM SPSS Statistics software Version 21.

### 2.5. Amplification of SIX Genes

The ten isolates identified as *Foa* were screened for the presence or absence of *SIX* genes using specific primers (Table 1) from those previously reported [13,19,21,33,34]. The amplification conditions included an initial denaturation step at 94 °C for 3 min, followed by 35 cycles. Each cycle consisted of 45 s of denaturation at 94 °C, 45 s of annealing at the appropriate temperatures (Table 2), a 45 s to 1 min extension at 72 °C, and a final 10 min extension at 72 °C. The size of the amplified product was compared to that reported in the literature. Negative (no DNA) and positive controls prepared with *F. oxysporum* f. sp. *lycopersici* race 1: CBS646.78 and race 2: CBS645.78 [19] were included in the reaction. Ten µL of the amplified product were separated by electrophoresis on a 2% agarose gel in TBE (Tris borate EDTA) buffer containing 0.5 µg/mL of ethidium bromide. The DNA was visualized under UV light.

## 3. Results

### 3.1. Foa Isolation and Molecular Detection

**Figure 1:**
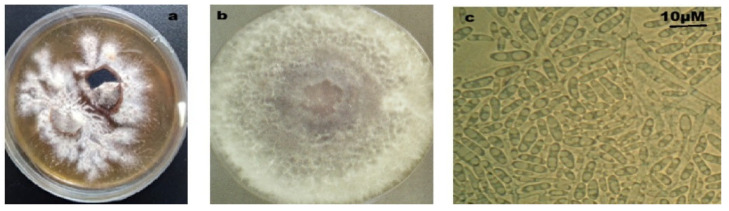
Isolation of *Foa* (*Fusarium oxysporum* f. sp. *albedinis*) from date palm rachis (**a**), its purification on PDA (**b**), and visualization under a light microscope (**c**).

The tested isolates were collected from three regions: Ouarzazate, Errachidia, and the Taznakht–Tata axis. They were purified and selected based on the macroscopic and microscopic characteristics typical of the *Fusarium* genus (Figure 1).

**Figure 2:**
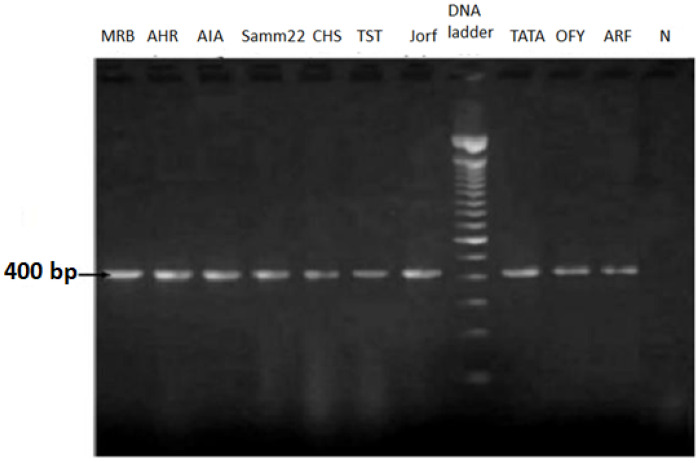
Ten isolates have an amplified product of 400 pb with the primer *Fot1*, confirming that the isolates were *Foa* (*Fusarium oxysporum* f. sp. *albedinis*); N: negative control.

Molecular detection using the *Foa*-specific primer pair FOA28 and TL3 (*Fot1*) enables the identification and differentiation of *Foa* isolates from other *Fusarium* species. The predicted 400 bp fragment was amplified in ten isolates from various localities of Moroccan oases locations (Figure 2). This confirmed that the isolates used in this study were *Foa*, the causative agent of date palm Bayoud disease. The ten isolates of *Foa* are presented in Table 2.

### 3.2. Aggressiveness Tests

**Figure 3:**
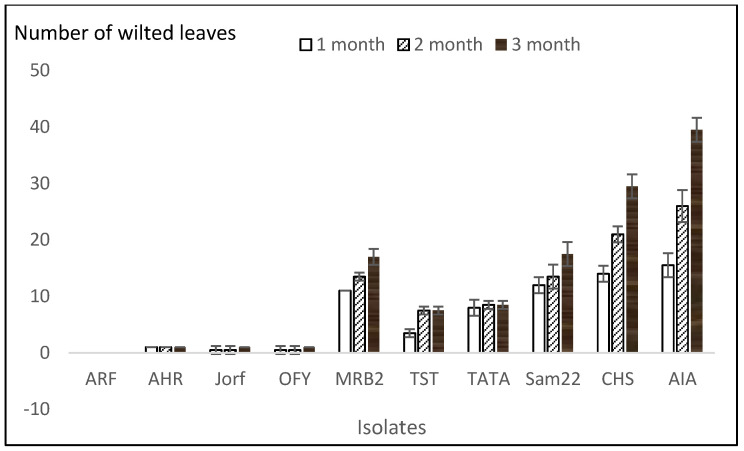
Number of wilted leaves in date palm Boufeggous seedlings inoculated with each *Foa* isolate.

**Figure 4:**
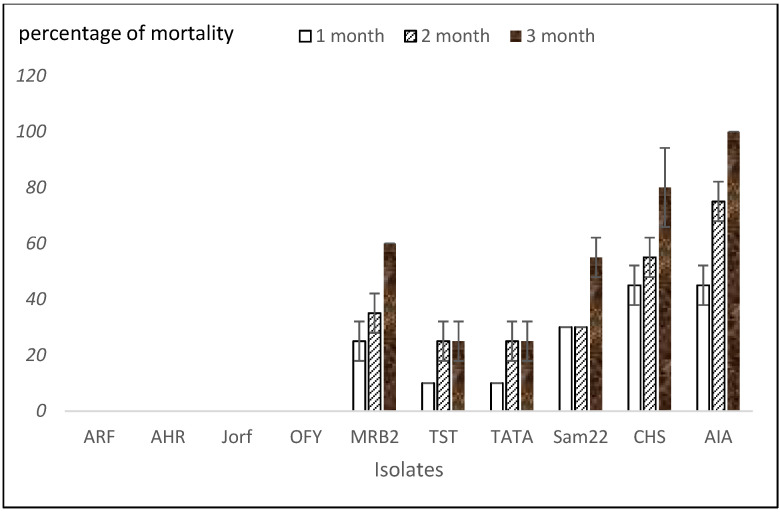
Percentage of mortality in date palm Boufeggous seedlings inoculated with each *Foa* isolate.

The results allowed for the ranking of the isolates based on their severity on date palm cultivar Boufeggous. Figure 3 and Figure 4 display the number of wilted leaves and the percentage of date palm seedling mortality per *Foa* isolate, respectively, at one, two, and three months post-inoculation. The figures indicate that the disease progress in date palm plants is influenced by both the *Foa* isolates and the time elapsed. With four isolates (ARF, AHR, Jorf, OFY), the percentage of mortality was nil, and the plants were practically symptom-free after three months post-inoculation, with less than two wilted leaves among the forty total leaves of the ten date palm plants tested. A necrotic zone was present at the site of *Foa* inoculation. These isolates were considered hypo-aggressive. For isolates TST and TATA, the mortality rate was approximately 25% three months post-inoculation. The number of wilted leaves and the percentage of mortality remained unchanged two months post-inoculation. These two isolates were considered moderately aggressive.

**Figure 5:**
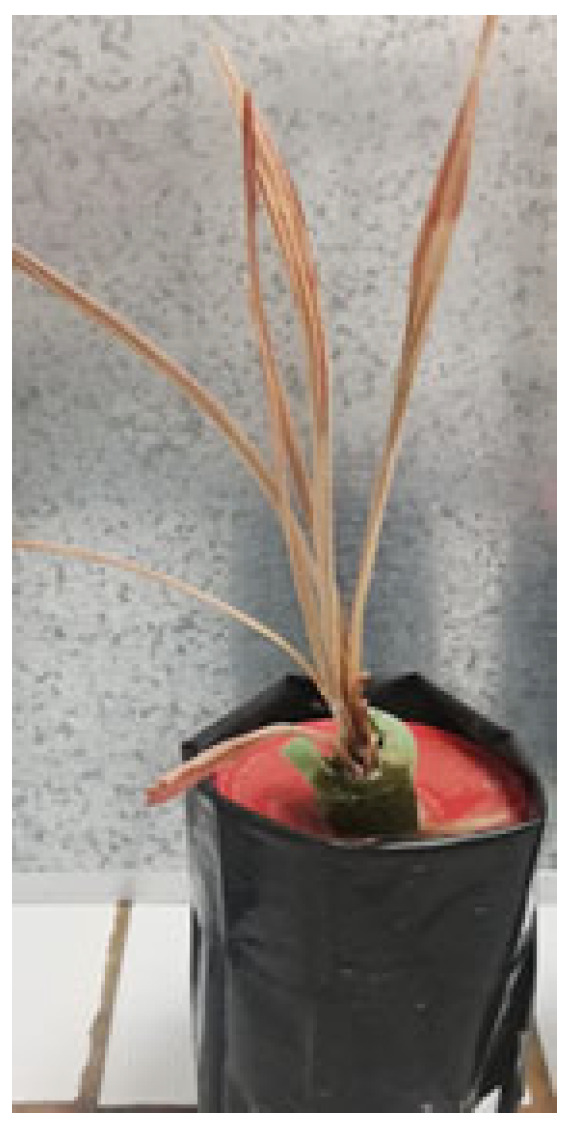
Date palm Boufeggous seedlings after inoculation with the re-isolated *Foa* (AIA).

The aggressive isolates (MRB2, Sam22, CHS, and AIA) showed a significant number of wilted leaves (ranging from 17 to 40 wilted leaves three months post-inoculation), with disease progression increasing from the first to the third month. AIA was the most aggressive isolate, followed by CHS and MRB2, which exhibited the same level of aggressiveness as the Samm22 isolate. The percentage of mortality was also significant, ranging from 55% for Sam22 to 100% for AIA three months post-inoculation. In addition, re-isolation of *Foa* from wilted leaves confirmed that it caused the observed symptoms (Figure 5). Moreover, the re-isolated fungus from wilted leaves was morphologically characterized and identified based on colony features and microscopic examination, which matched the characteristics of the originally inoculated *Foa* isolate. Furthermore, molecular confirmation using specific primers targeting *Foa* was performed to ensure that the re-isolated organism was indeed *Foa*.

### 3.3. Relationship Between Aggressiveness and SIX Genes in Foa

The distribution of SIX genes in *Foa* is presented in Table 3.

The profile of *SIX* genes varied depending on the isolates’ aggressiveness. The aggressive isolates exhibited a high number of *SIX* genes, with 9 genes for AIA, 10 genes for MRB2, 11 genes for both CHF, and 12 genes for Sam22, compared to the hypo-aggressive isolates, which had fewer *SIX* genes (0 genes for Jorf2, 3 genes for both OFY and AHR, and 4 genes for ARF). However, the number of *SIX* genes cannot be a reliable indicator of aggressiveness, as determined by the TST isolate, which possesses only three SIX genes but showed more Bayoud disease symptoms than ARF and AHR, which have four and three SIX genes, respectively.

**Figure 6:**
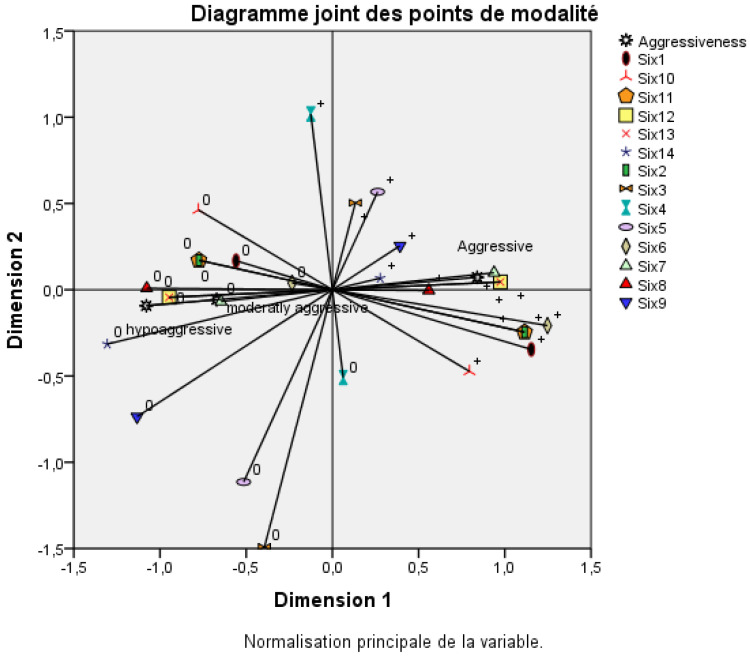
Multiple correspondence analysis (MCA) showing the correlation between aggressiveness and the detected *SIX* genes. 0: absence of *SIX* gene, +: presence of *SIX* gene.

The multiple correspondence analysis (MCA) revealed a positive correlation between *SIX* genes 1, 2, 6, 7, 8, 11, 12, and 13 on one side and the aggressive isolates on the other (Figure 6). Interestingly, SIX genes 12 and 13 were detected in all four aggressive isolates but absent in isolates from other categories. The *SIX* genes 1, 2, 6, 11, and 7 were not detected in the hypo-aggressive and moderately aggressive isolates, but they appeared in the aggressive isolates as follows: *SIX6* in Sam22; *SIX1* in MRB and Sam22; *SIX11* and *SIX2* in MRB2, Sam22, and CHS; and *SIX7* in MRB2, CHS, and AIA. AIA was the most aggressive isolate; all its *SIX* genes were shared with the other aggressive isolates except for the *SIX4* gene. However, this gene was also found in hypo-aggressive and moderately aggressive isolates (OFY and TATA). In addition, the SAM22 and CHS isolates were aggressive but did not have the *SIX4* gene. Moreover, the unique difference between the CHS and Sam22 isolates was that the *SIX6* gene in Sam22 was replaced by *SIX7* in CHS. Still, both isolates were aggressive. This was confirmed with the AIA isolate, which did not have the *SIX 6* gene even though it was the most aggressive isolate. The *SIX8* gene was present in the four aggressive isolates (MRB, SAM22, CHS, and AIA) described here but was also shared with some less aggressive isolates (AHR and TATA). From this study, we concluded that many *SIX* genes were associated with aggressive *Foa* isolates. Three different combinations of *SIX* genes could be linked to aggressiveness: the combination *SIX* genes 2, 7, 8, 11, 12, and 13 found in the MRB2 and CHS isolates; the combination *SIX* genes 2, 6, 8, 11, 12, and 13 in the Sam22 isolate; and the combination *SIX* genes 4, 7, 8, 12, and 13 in the AIA isolate. Of all these combinations, *SIX* genes 12 and 13 were the only genes present in all the aggressive isolates and absent in all the hypo-aggressive or moderately aggressive isolates.

**Figure 7:**
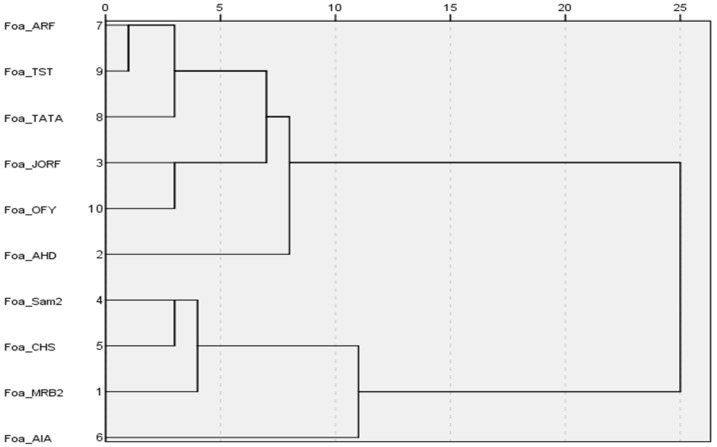
Hierarchical tree using the average distance between the classes based on the distribution of *SIX* genes in *Foa* isolates. The class combination distance has been rescaled.

Hypo-aggressive and moderately aggressive isolates seemed to have fewer *SIX* genes than the aggressive isolates did. In addition, all these genes were also found in the aggressive isolates. Moreover, the hierarchical tree relying on the correlation between *SIX* genes and the isolates (Figure 7) allowed for the grouping of the isolates into two clusters, the first clade containing all the isolates with high levels of aggressiveness (MRB2, Sam22, CHS, and AIA) and the second clade with hypo-aggressive and moderately aggressive isolates.

## 4. Discussion

This study is the first to show a positive correlation between the presence of *SIX* genes and aggressiveness in the *Foa*-date palm pathosystem. The aggressiveness was monitored at one, two, and three months post-inoculation. The disease severity could be classified into three categories depending on the *Foa* isolates. The disease symptoms started to look bad within the first month for the aggressive isolates and continued progressing gradually until the plants wilted completely. These symptoms reflect the massive tissue colonization by the pathogen, which obstructs the xylem vessels and prevents rise in the sap towards the aerial parts, leading to date palm death. This colonization was confirmed by reisolating *Foa* from the leaves, although the inoculation was started on the roots. Such infection indicated the failure of date palm’s defense against this pathogen [5]. As for the moderately aggressive isolates, the symptoms were less severe; they appeared in the first month but stopped progressing from the second month, which reflected the ending of Bayoud disease advancement. This could be associated with a successful date palm defense reaction, which occurred earlier and more rapidly than with aggressive isolates, leading to almost asymptomatic plants three months after *Foa* inoculation. Similar results have been reported for AHR, a hypo-aggressive isolate, infecting date palm seedlings [6,7].

The involvement of *SIX* genes in virulence has been reported regarding several *F. oxysporum* species, such as *F. oxysporum* f. sp. *lycopersici* [13,18] and *F. oxysporum* f. sp. *cepae* [21]. Here, we screened ten isolates from infected date palm rachis for the presence/absence of *SIX* gene homologs. All the isolates had the transposable element Fot1, confirming that these isolates were effectively *Foa* [31,32]. The results showed that *Foa* isolates harbored several *SIX* genes distributed differently depending on the isolates. This suggests that *Foa*, like other *F. oxysporum* species, has accessory chromosomes that carry *SIX* genes in addition to the core chromosomes responsible for saprophytic life [8]. Gene deletion experiments have previously demonstrated the involvement of *SIX* genes in virulence. Deletion of each of *SIX1*, *SIX3*, *SIX5*, or *SIX6* gene significantly reduced the virulence of *F. oxysporum* f. sp. *lycopersici* in tomato [11,16,17,18]. The profiles of *SIX* genes in *Foa* convincingly distinguished between the aggressive and less aggressive isolates. The less aggressive isolates had fewer *SIX* genes (between 0 and 6 SIX genes) than the aggressive isolates (between 9 and 11 *SIX* genes). Similar results were previously reported in *F. oxysporum* f. sp. *cubense* the causal agent of Panama disease in banana. In this case, the tropical Race 4 was reported to be a devasting race that harbors many *SIX* genes [35,36]. Taylor et al. (2016) [21] differentiated highly virulent isolates of *F. oxysporum* f. sp. *cepae* from those with low virulence according to the abundance of *SIX* genes. They confirmed that the isolates with reduced virulence had fewer *SIX* genes, while highly virulent isolates had many more *SIX* genes. This observation cannot be generalized because the TST isolate has just 3 *SIX* genes and showed more Bayoud disease symptoms than ARF, which has 4 *SIX* genes. Additionally, the number of *Foa* isolates used here is small (ten isolates), and to confirm this conclusion, we need to increase the number of *Foa* isolates.

With the less aggressive isolates, a necrotic zone developed at the *Foa* inoculation site, but Bayoud symptoms were attenuated, and the pathogen progression was restricted. Such a response could not only be attributed to *SIX* genes since the isolate Jorf2 was devoid of the *SIX* genes but still presented some symptoms. In addition, all the *SIX* genes identified in the less aggressive isolates were also present in the aggressive isolates that developed Bayoud disease on date palm plants.

The multiple correspondence analysis (Figure 6) positively correlated the *SIX* genes 2, 6, 7, 8, 11, 12, and 13 on one side and aggressiveness on the other side. All these *SIX* genes were present only in the aggressive isolates except the *SIX8* gene, which was shared between aggressive and less aggressive isolates. This is in line with the work of Kotera et al.’s (2022) [37] work that differentiated pathogenic isolates of *F. oxysporum* f. sp. *pisi* (*Fop*), the causal agent of pea wilt disease, based on the distribution of its *SIX* genes. The pathogenic isolates carried *SIX6* and/or *SIX13* genes, while the non-pathogenic isolates did not. In addition, Sasaki et al. (2015), based on the rDNA intergenic spacer, grouped 20 *F. oxysporum* f. sp. *cepae* isolates into one clade; all the isolates belonging to this group possessed *SIX3*, *SIX5*, and *SIX7* genes and were pathogenic to onion seedlings [38]. Lievens et al. (2009) suggested that *SIX6* and SIX7 genes potentially influence the pathogenicity of *F. oxysporum* f. sp. *lycopersici* towards tomatoes [19]. The *SIX6* gene was also reported for *F. oxysporum* f. sp. *radicis-cucumerinum* virulence against cucurbit species [10].

In our study, *SIX12* and *SIX13* were found in all the aggressive isolates (four isolates). They were absent in the other isolates belonging to the other categories, suggesting a potential role in *Foa* aggressiveness for these genes. AIA is the most aggressive isolate, and all its *SIX* genes were shared with the other aggressive isolates except for the *SIX4* gene. In addition, SAM22 and CHS isolates were aggressive but did not have the *SIX4* gene.

If the *SIX8* gene contributes to aggressiveness, it needs to be combined with other *SIX* genes. Thus, it seems that aggressiveness cannot be controlled by a unique *SIX* gene in *Foa* but can rather be affected by different combinations of *SIX* genes, depending on the isolate.

Furthermore, the genome of *Foa* has been sequenced for four isolates (isolate 9, 13116, *Foa* 133, *Foa* 44) and compared to the well-characterized tomato pathogenic *Fol* [29,39,40,41]. This comparison led to the reporting of five homologs (*SIX1*, *SIX6*, *SIX9*, *SIX11*, and *SIX14*) exhibiting higher to lower sequence identity to *Fol* (97.3% for *SIX11*, 91.3% for *SIX9*, 83.4% for *SIX1*, 77.5% for *SIX6*, 68.2% for *SIX14*) [40]. In addition, NGS of *Foa* isolates enabled a preliminary survey of candidate virulence loci, identifying *SIX* genes predicted to encode effectors [29,40,41]. In our study, PCR assays targeting homologous *SIX* genes uncovered the same six genes, *SIX1*, *SIX5*, *SIX6*, *SIX9*, *SIX11*, and *SIX14*, across diverse isolates and additionally revealed eight further *SIX* genes of potential pathogenic relevance. Our data showed that the assessments based solely on draft genome assemblies might carry inherent uncertainties, because the apparent absence of a *SIX* gene may reflect assembly gaps rather than true gene loss. Only a gap-free, high-quality reference genome can definitively confirm absence. Nevertheless, to link *SIX* genes to aggressiveness, loss-of-function mutants of *SIX* genes need to be developed using either CRISPR-Cas9 or other means.

Although our study focused on the presence/absence of selected *SIX* genes in *Fusarium oxysporum* f. sp. *albedinis* isolates, it is important to note that the presence of these genes alone does not provide information about their sequence variation, evolutionary dynamics, or functional divergence. Future studies involving the sequencing of individual *SIX* genes across diverse *Foa* isolates, followed by bioinformatic analysis, would be valuable for identifying sequence polymorphisms, conserved motifs, or lineage-specific adaptations. Such analyses could improve our understanding of how *SIX* gene evolution contributes to host specificity, virulence, and the emergence of aggressive strains. In addition, this would provide a foundation for developing molecular markers for pathogen surveillance and early detection strategies in date palm cultivation. Moreover, several studies (e.g., [11,28]) have shown that *SIX* gene expression is tightly regulated and can vary depending on host interactions, environmental conditions, or the developmental stage of infection. This addition emphasizes the need for integrating comparative genomics, transcriptomics, and precise functional genetics to validate the role of *SIX* genes in *Foa* aggressiveness and host specificity.

## 5. Conclusions

The findings of this study establish a strong correlation between the presence of specific *SIX* genes and the aggressiveness of *Foa* isolates responsible for Bayoud disease in date palms. Aggressive isolates harbored a greater number of *SIX* genes compared to hypo-aggressive and moderately aggressive isolates, with *SIX12* and *SIX13* being consistently associated with high levels of aggressiveness. However, this study also highlights that the number of *SIX* genes alone is not a definitive indicator of aggressiveness, as observed in the TST isolate, which induced severe symptoms despite possessing only three *SIX* genes. Furthermore, different combinations of *SIX* genes were identified among aggressive isolates, suggesting that multiple genes contribute collectively to virulence rather than a single determinant. These results support the hypothesis that *Foa*, like other *F. oxysporum* species, possesses accessory chromosomes carrying *SIX* genes that play a crucial role in pathogenicity. Nevertheless, the presence of *SIX* genes alone does not fully explain the variation in aggressiveness, as host–pathogen interactions and other genetic factors may also influence disease severity. Expanding this study to a larger set of *Foa* isolates and completing functional analysis of the prominent *SIX* genes through gene knockout and complementation would provide further insights into the genetic determinants of virulence and help refine our understanding of the molecular mechanisms underlying Bayoud disease progression.

## Figures and Tables

**Table 1:** Primer pairs used to detect (SIX) genes in Xylem secretions.

Primer	Sequence (5′-3′)	Target	Expected Fragment Size (bp)	Annealing Temperature	Sources
P12-F1	GTATCCCTCCGGATTTTGAGC	*SIX1*	992	60 °C	[34]
P12-R1	AATAGAGCCTGCAAAGCATG				[13]
SIX2-F2	CAACGCCGTTTGAATAAGCA	*SIX2*	749	55.6 °C	[34]
SIX2-R2	TCTATCCGCTTTCTTCTCTC				
SIX3-F1	CCAGCCAGAAGGCCAGTTT	*SIX3*	608	55.5 °C	[34]
SIX3-R2	GGCAATTAACCACTCTGCC				
SIX4-F1	TCAGGCTTCACTTAGCATAC	*SIX4*	967	55.5 °C	[19]
SIX4-R2	GCCGACCGAAAAACCCTAA				
SIX5-F1	ACACGCTCTACTACTCTTCA	*SIX5*	667	59 °C	[19]
SIX5-R2	GAAAACCTCAACGCGGCAAA				
SIX6-F1	CTCTCCTGAACCATCAACTT	*SIX6*	793	56 °C	[19]
SIX6-R2	CAAGACCAGGTGTAGGCATT				
SIX7-F1	CATCTTTTCGCCGACTTGGT	*SIX7*	862	58 °C	[19]
SIX7-R2	CTTAGCACCCTTGAGTAACT				
SIX8-F1	TCGCCTGCATAACAGGTGCCG	*SIX8*	250	59 °C	[33]
SIX8-R2	TTGTGTAGAAACTGGACAGTCGATGC				
SIX9-F1	GGCCCAGCCCTAGTCTAACTCC	*SIX9*	458	69 °C	[21]
SIX9-R2	AACTTAACATGCTGGCCGTCAATCG				
SIX10-F1	GTTAGCAACTGCGAGACACTAGAA	*SIX10*	636	65 °C	[21]
SIX10-R2	AGCAACTTCCTTCCTCTTACTAGC				
SIX11-F1	ATTCCGGCTTCGGGTCTCGTTTAC	*SIX11*	559	61 °C	[21]
SIX11-R2	GAGAGCCTTTTTGGTTGATTGTAT				
SIX12-F1	CTAACGAAGTGAAAAGAAGTCCTC	*SIX12*	449	61 °C	[21]
SIX12-R2	GCCTCGCTGGCAAGTATTTGTT				
SIX13-F1	CCTTCATCRTCGACAGTACAACG	*SIX13*	1027	61 °C	[21]
SIX13-R2	ATCAAACCCGTAACTCAGCTCC				
SIX14-F1	ATAAAGTGCGACTGGACTTCTGCC	*SIX14*	449	67 °C	[21]
SIX14-R2	ACCCCCATCCACATTCCTAAGCGA				

**Table 2:** *Foa* (*Fusarium oxysporum* f. sp. *albedinis)* isolates used in this study.

Region	Locality	Isolate
Ouarzazate	Ahardane	AHR
Errachidia	Oufous Youssef	OFY
	Jorf	JRF
	Sam2	Sam2
	Cheikh Sifa	CHS
	Arfoud	AOD
Taznakht-Tata Axis	Ait Aissa	AIA
	Mrabet2	MRB2
	Tissn’t	TST
	Tata	TATA

**Table 3:** Relationship between aggressiveness and SIX genes profile for 10 *Foa* isolates.

Species	Isolate	*Fot* *1*	Aggressiveness *	*SIX* *1*	*SIX* *2*	*SIX* *3*	*SIX* *4*	*SIX* *5*	*SIX* *6*	*SIX* *7*	*SIX* *8*	*SIX* *9*	*SIX* *10*	*SIX* *11*	*SIX* *12*	*SIX* *13*	*SIX* *14*
*Foa*	MRB2	+	++	+	+	−	−	−	−	+	+	+	+	+	+	+	+
*Foa*	AHD	+	−	−	−	−	−	−	−	−	+	−	+	−	−	−	+
*Foa*	Jorf2	+	−	−	−	−	−	−	−	−	−	−	−	−	−	−	−
*Foa*	SAM22	+	++	+	+	+	−	+	+	−	+	+	+	+	+	+	+
*Foa*	CHS	+	++	−	+	+	−	+	−	+	+	+	+	+	+	+	+
*Foa*	AIA	+	++	−	−	+	+	+	−	+	+	+	−	−	+	+	+
*Foa*	ARF	+	−	−	−	+	−	+	−	−	−	+	−	−	−	−	+
*Foa*	TATA	+	+	−	−	+	+	+	−	−	+	+	−	−	−	−	+
*Foa*	TST	+	+	−	−	+	−	−	−	−	−	+	−	−	−	−	+
*Foa*	OFY	+	−	−	−	+	+	+	−	−	−	−	−	−	−	−	−
*Fol*	*Fol* race1 CBS646.78	−	NT	+	+	+	+	+	+	+	+	+	+	+	+	+	+
*Fol*	*Fol* race2 CBS645.78	−	NT	+	+	+	−	+	+	+	+	+	+	+	+	+	+

* Aggressiveness, −: hypo-aggressive, +: moderately aggressive, ++: aggressive, NT: not tested.

## Data Availability

Data are contained within the article and Supplementary Materials.

## Supplementary Materials

The following supporting information can be downloaded at: https://www.mdpi.com/article/10.3390/plants14111721/s1, Figure S1: Sites (*) of *Fusarium oxysporum albedinis* isolation in Morocco; Figure S2: 12-month-old date palm seedlings of BFG cultivar in flasks containing Hoagland solution after inoculation with different *Foa* isolates.
