# Supplementary material for: “Secreted in Xylem” Genes (SIX Genes): Relationship to the Aggressiveness of Fusarium oxysporum f. sp. albedinis
Source: Plants (Basel). 2025 Jun 5;14(11):1721. doi: 10.3390/plants14111721 (PMC12158221; doi:10.3390/plants14111721)
Supplement: Supplementary file 1 [file plants-14-01721-s001.zip › plants-3600858-supplementary.pdf]

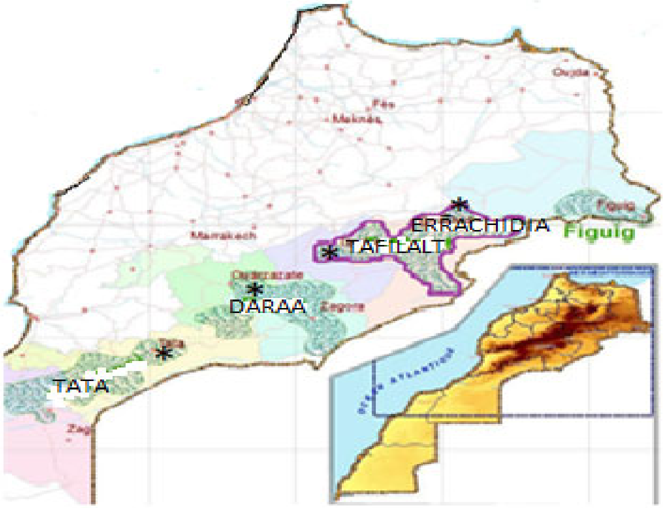


**Figure S1.** Sites (*) of *Fusarium oxysporum albedinis* isolation in Morocco.

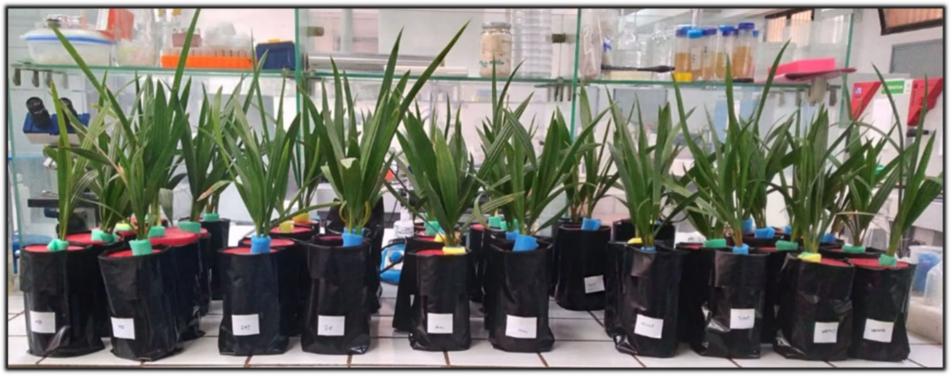

**Figure S2.** 12-month-old date palm seedlings of BFG cultivar in flasks containing Hoagland solution after inoculation with different *Foa* isolates.
